# Long noncoding RNA KCNQ1OT1 inhibits osteoclast differentiation by regulating the miR-128-3p/NFAT5 axis

**DOI:** 10.18632/aging.204088

**Published:** 2022-05-19

**Authors:** Hengshuo Zhang, Lu Chen, Ziyu Wang, Zhenqian Sun, Yu Shan, Qinghui Li, Linzeng Qi, Hongliang Wang, Yunzhen Chen

**Affiliations:** 1 Department of Orthopedics, Qilu Hospital of Shandong University, Jinan, Shandong 250012, PR China; 2 Cheeloo College of Medicine, Shandong University, Jinan, Shandong 250012, PR China

**Keywords:** osteoporosis, osteoclast differentiation, long noncoding RNA KCNQ1OT1, microRNA-128-3p, NFAT5

## Abstract

Noncoding RNAs play an important role in regulating osteoclast differentiation. We investigated whether and how potassium voltage-gated channel subfamily Q member 1 overlapping transcript 1 (KCNQ1OT1), a long noncoding RNA, regulates osteoclast differentiation. We found that the expression of KCNQ1OT1 was downregulated in osteoporotic bone tissue. Then transfection of KCNQ1OT1 overexpression vectors or small interfering RNAs showed that the proliferation, migration, and osteoclast differentiation of RAW 264.7 cells were inhibited by KCNQ1OT1 upregulation, while they were promoted by KCNQ1OT1 knockdown. Interestingly, we found and confirmed that miR-128-3p was a target of KCNQ1OT1 using online databases, dual luciferase reporter assays and quantitative real-time polymerase chain reaction, and that it inhibited the expression of miR-128-3p. Moreover, we confirmed that miR-128-3p directly targeted nuclear factor of activated T cell 5 (NFAT5), a protein that combines with osteoprotegerin and thus regulates osteoclastogenesis with the presence of the receptor activator of nuclear factor κB ligand. Furthermore, we demonstrated that both the knockdown of KCNQ1OT1 and the overexpression of miR-128-3p attenuate the expression of NFAT5, while upregulating the osteoclastogenesis markers c-Fos, NFATc1, and Ctsk. The results from overexpression of KCNQ1OT1 and the inhibition of miR-128-3p were contrary to the above. Finally, we found that the inhibition of osteoclast differentiation by KCNQ1OT1 overexpression could be rescued using a miR-128-3p mimic, while the enhancement of migration and osteoclast differentiation by si-NFAT5 could be reversed with a miR-128-3p inhibitor. These results suggested that KCNQ1OT1 regulates the osteoclast differentiation via the miR-128-3p/NFAT5 axis.

## INTRODUCTION

Osteoporosis (OP) is a general metabolic bone disease that is characterized by a decrease in bone mass and density, degeneration of bone microstructure and an increase in bone brittleness [1]. In recent years, OP has become a growing public health concern and is responsible for a stressful burden on patients’ families as well as society as a whole [2]. Bone homeostasis is the dynamic balance of bone formation by osteoclasts and resorption by osteoclasts, and disorders of this balance due to insufficient bone formation or excessive bone resorption will cause osteoporosis [1, 3]. However, the molecular mechanism driving osteoblast and osteoclast differentiation remains to be elucidated.

Long noncoding RNAs (lncRNAs) are a type of transcribed RNA composed of more than 200 nucleotides, which are located in the nucleus or cytoplasm [4–6]. They play an important role in the processes of proliferation, apoptosis and differentiation [7–9]. Some lncRNAs have been shown to be related to osteoclast differentiation [10–14]. It has been demonstrated that as a competing endogenous RNAs (ceRNAs), lncRNAs regulate the expression level of target genes through sponging microRNAs (miRNAs) [15–17].

Potassium voltage-gated channel subfamily Q member 1 overlapping transcript 1 (KCNQ1OT1) is located in chromosome 11 and plays an important role in the regulation of osteogenic differentiation [18]. Recently, Fei et al., reported a decreased expression of KCNQ1OT1 in male patients with osteoporosis [19]. KCNQ1OT1 has been shown to play an important role in osteogenesis, and can accelerate fracture healing by promoting the proliferation, migration, and survival of osteoblasts [20]. Meanwhile, KCNQ1OT1 can promote the osteogenic differentiation of human bone marrow mesenchymal stem cells (hBMSCs) and mouse mesenchymal stem cells (mMSCs) [21, 22]. Furthermore, overexpression of KCNQ1OT1 can upregulate the expression of IL-10 and reverse the macrophage polarization induced by polymethylmethacrylate via regulating the expression of miR-21a-5p in RAW 264.7 cells, to improve osteolysis [23]. However, there have been a few studies on the effects of KCNQ1OT1 on osteoclasts and the corresponding mechanism is also needs to be further explored.

miRNAs are small, noncoding RNAs of only 18–24 nucleotides. To date, numerous miRNAs have been shown to take part in the process of osteoblast and osteoclast differentiation [24, 25]. miR-128-3p has been widely studied in glioma, osteosarcoma and lung cancer [26–28], and it can also delay bone fracture healing [29]. Nuclear factor of activated T cell 5 (NFAT5) is an osmoprotective transcription factor belonging to the Rel family of transcription factors (TFs), which is involved in protecting cells from hypertonic stress [30]. Liao et al., [31] demonstrated that NFAT5 promotes cementoblast differentiation. In addition, Agnes Schroder et al., found that NFAT5 could bind to the promoter region of osteoprotegerin (OPG), resulting in upregulation of OPG expression to inhibit the osteoclastogenesis induced by receptor activator of nuclear factor κB ligand (RANKL) [32]. Nevertheless, studies on the mechanism of miR-128-3p in osteoclast differentiation are still incomplete.

In this study, we focused on the role of the KCNQ1OT1/miR-128-3p/NFAT5 axis in regulating RAW 264.7 cell differentiation and clarified the corresponding mechanism.

## RESULTS

### KCNQ1OT1 suppresses the proliferation and migration of RAW 264.7 cells

**Figure 1:**
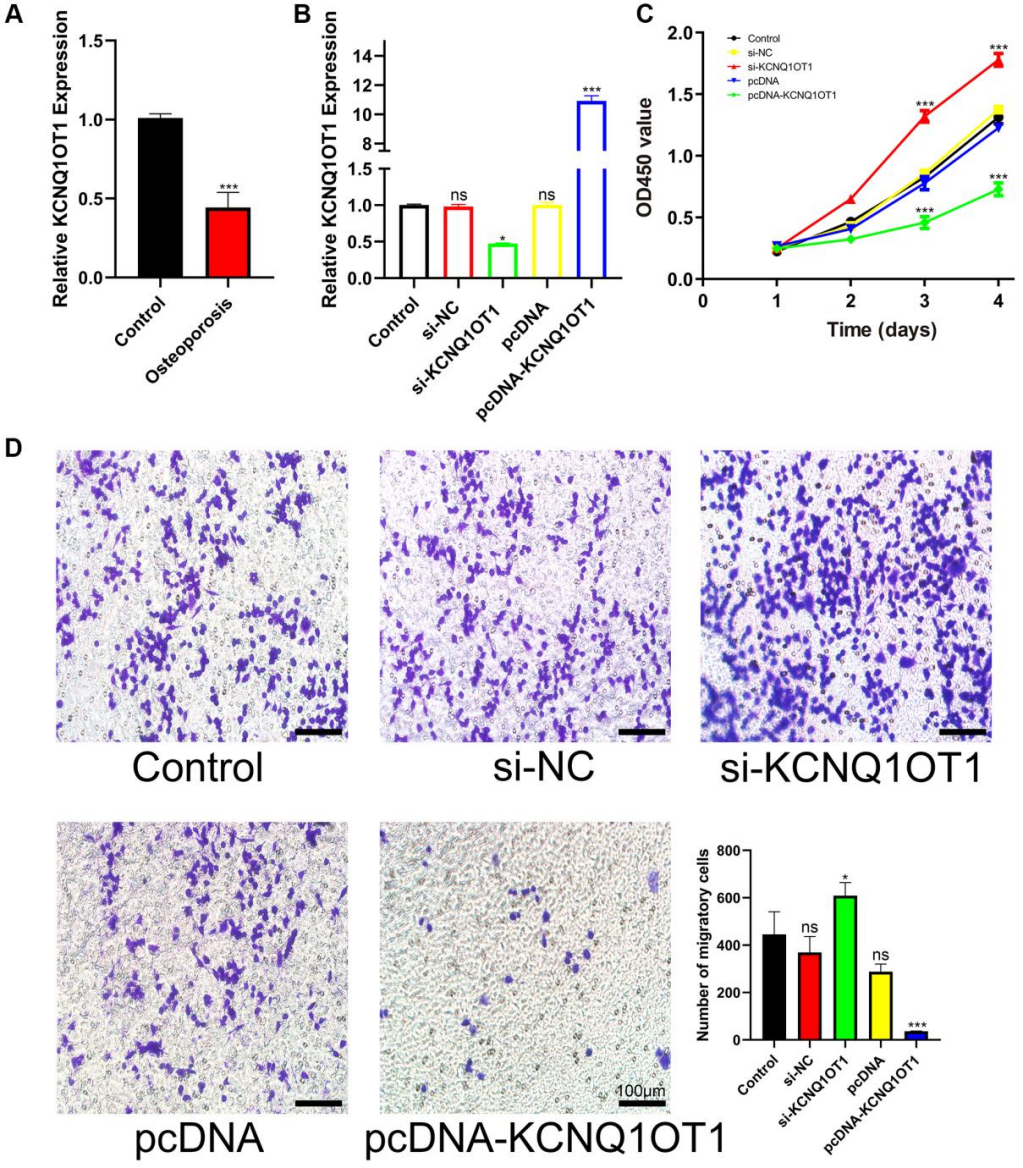
**KCNQ1OT1 suppresses the proliferation and migration of RAW 264.7 cells.** (**A**) qRT-PCR for relative expression of KCNQ1OT1 in six bone tissues (three osteoporotic and three non-osteoporotic). (**B**) Relative expression of KCNQ1OT1 in RAW 264.7 cells transfected with pcDNA-KCNQ1OT1, si-KCNQ1OT1 or control vectors, as measured by qRT-PCR. (**C**) The proliferation curve and (**D**) transwell migration assays of RAW 264.7 cells transfected with pcDNA-KCNQ1OT1, si-KCNQ1OT1 or corresponding control vectors (Scale bar: 100 μm). ^*^P < 0.05, ^***^P < 0.001, ns: not significant.

The expression of KCNQ1OT1 was measured by quantitative real-time polymerase chain reaction (qRT-PCR). Compared with healthy controls, the expression of KCNQ1OT1 in osteoporotic bone tissue was significantly downregulated (Figure 1A). To investigate the effect of KCNQ1OT1 on the proliferation and migration of RAW 264.7 cells, a KCNQ1OT1 overexpression vector and KCNQ1OT1 siRNAs were transfected into RAW 264.7 cells, respectively (Figure 1B). Cell counting kit (CCK)-8 assays showed that overexpression of KCNQ1OT1 significantly suppressed the proliferation of RAW 264.7 cells, while downregulating KCNQ1OT1 expression promoted cell proliferation (Figure 1C). Transwell migration assays were also conducted to determine the effect of KCNQ1OT1 on RAW 264.7 cell migration (Figure 1D). Our results indicated that KCNQ1OT1 inhibited the proliferation and migration of RAW 264.7 cells.

### KCNQ1OT1 inhibits the osteoclast differentiation of RAW 264.7 cells

**Figure 2:**
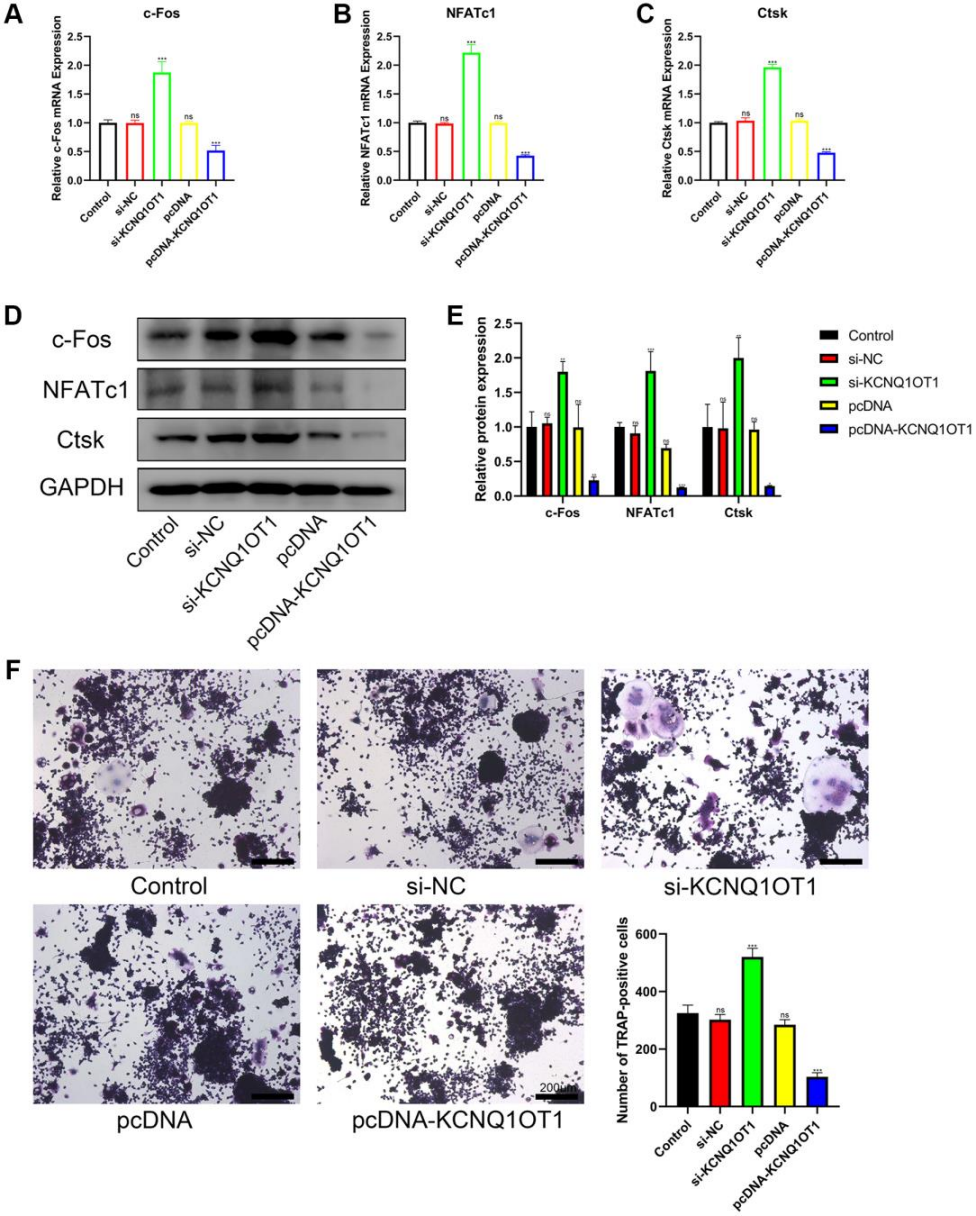
**KCNQ1OT1 inhibits the osteoclast differentiation of RAW 264.7 cells.** (**A**–**E**) RAW 264.7 cells transfected with pcDNA-KCNQ1OT1, si-KCNQ1OT1, or corresponding control vectors and treated with RANKL for 5 days, the mRNA and protein levels of the osteoclastogenesis markers c-Fos, NFATc1, and Ctsk were measured by qRT-PCR and Western blotting. (**F**) A Tartrate-resistant acid phosphatase (TRAP) staining kit was used for osteoclast staining, and TRAP-positive multinucleated cells were counted (Scale bar: 200 μm). ^*^P < 0.05, ^**^P < 0.01; ^***^P < 0.001, ns: not significant.

To assess the importance of KCNQ1OT1 in osteoclast differentiation, a KCNQ1OT1 overexpression vector or KCNQ1OT1 siRNAs were transfected into RAW 264.7 cells, followed by stimulation with RANKL (100 ng/ mL) for 5 days to induce osteoclast differentiation. After 5 days, qRT-PCR and Western blotting results showed that KCNQ1OT1 could decrease the expression of osteoclastogenesis markers such as c-Fos, NFATc1, and Ctsk, while the results from the KCNQ1OT1 knockdown group were opposite (Figure 2A–2E). We also found that overexpression of KCNQ1OT1 significantly reduced the number of Tartrate-resistant acid phosphatase (TRAP)-positive multinucleated osteoclasts by TRAP staining, while the result was opposite after KCNQ1OT1 downregulation (Figure 2F). The above results indicated that KCNQ1OT1 plays an inhibitory role in osteoclast differentiation.

### KCNQ1OT1 directly targets miR-128-3p

**Figure 3:**
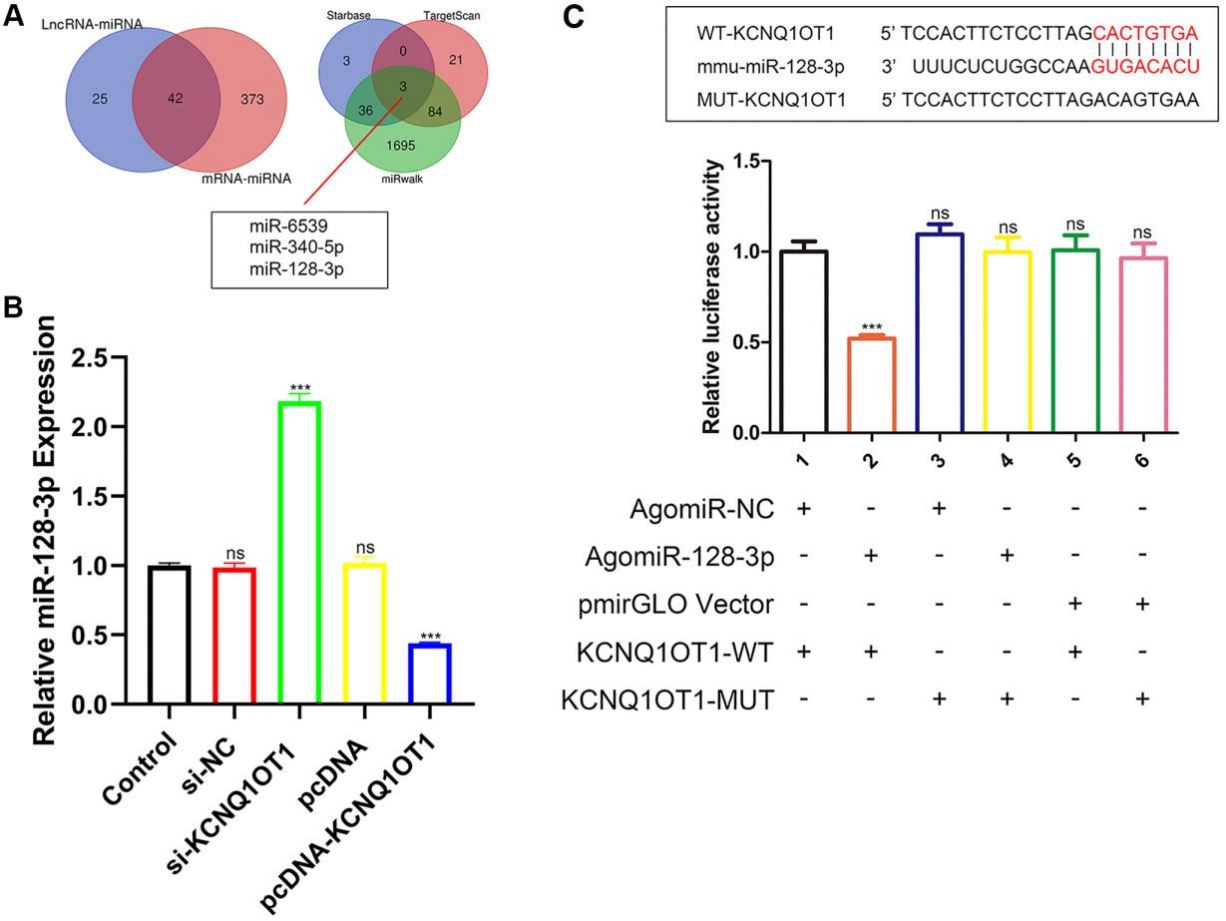
**KCNQ1OT1 directly targets miR-128-3p.** (**A**) StarBase, miRwalk and TargetScan databases showing that miR-128-3p might be a potential target of KCNQ1OT1. (**B**) Relative expression of miR-128-3p in RAW 264.7 cells transfected with pcDNA-KCNQ1OT1, si-KCNQ1OT1, or corresponding control vectors. (**C**) StarBase database was used to predict the binding site between KCNQ1OT1 and miR-128-3p. Dual-luciferase reporter assays on HEK-293T cells transfected with agomiR-NC or agomiR-128-3p in wild-type (WT) and mutant-type (MUT) groups. ^***^P < 0.001, ns: not significant.

lncRNAs can adsorb miRNAs as a sponge to reduce their effect on downstream target genes. In order to explore the downstream mechanism of KCNQ1OT1, starBase was used to predict the potential miRNA targets of KCNQ1OT1, and 67 related miRNAs were predicted. The miRNAs that might regulate the expression of NFAT5 were also predicted by starBase, and 415 miRNAs were identified. After generating a Venn diagram, 42 related miRNAs were selected for further analysis. Subsequently, miRNAs regulating NFAT5 were predicted by miRwalk and TargetScan respectively, among which 108 miRNAs were predicted using TargetScan and 1818 miRNAs were predicted using miRwalk. After comparing these with the 42 miRNAs predicted by starBase, as can be seen in Figure 3A, three miRNAs were identified: miR-6539, miR-340-5p and miR-128-3p. Among them, miR-128-3p has been reported to play an inhibitory role in the process of fracture healing, and its overexpression can inhibit the apoptosis of RAW 264.7 cells. Moreover, a highly conserved binding sequence was found between KCNQ1OT1 and miR-128-3p. We further performed dual luciferase assays to verify the relationship between KCNQ1OT1 and miR-128-3p. As a result, the miR-128-3p agomir significantly reduced the luciferase activity of HEK-293T cells in the KCNQ1OT1-WT group, while we did not observe significant changes in the luciferase activity of the KCNQ1OT1-MUT group (Figure 3C). In addition, qRT-PCR showed that KCNQ1OT1 overexpression inhibited the expression of miR-128-3p in RAW 264.7 cells, while KCNQ1OT1 knockdown enhanced it (Figure 3B). In conclusion, the above results indicated that KCNQ1OT1 acted as a sponge for miR-128-3p.

### miR-128-3p promotes the viability and osteoclast differentiation of RAW 264.7 cells

**Figure 4:**
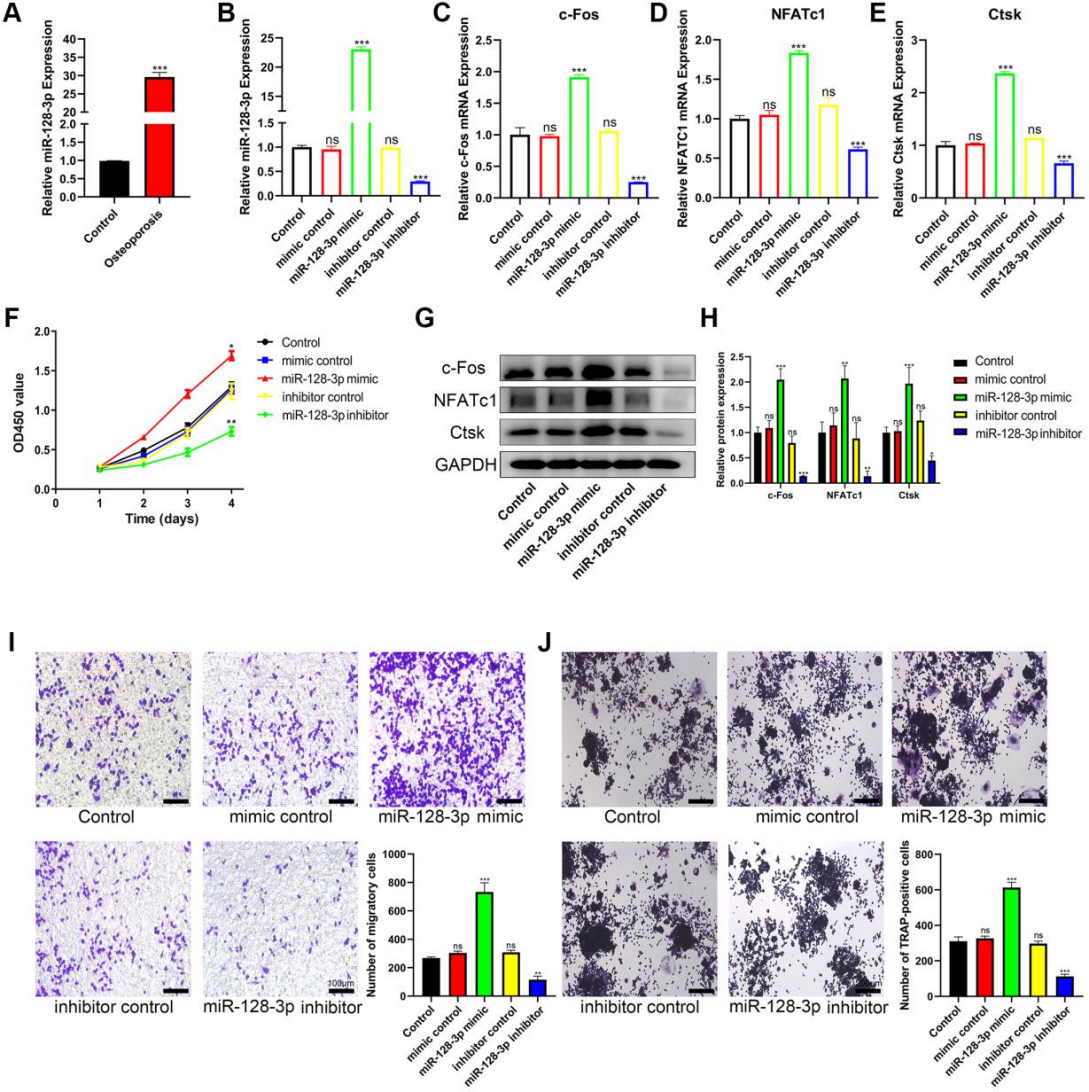
**miR-128-3p promotes the viability and osteoclast differentiation of RAW 264.7 cells.** (**A**) qRT-PCR for relative expression of miR-128-3p in bone tissues (three non-osteoporotic and three osteoporotic). (**B**) RAW 264.7 cells were transfected with miR-128-3p mimic or miR-128-3p inhibitor, respectively. The expression levels of miR-128-3p were measured by qRT-PCR. (**C**–**E**) The mRNA levels of c-Fos, NFATc1, and Ctsk were analyzed by qRT-PCR, respectively. (**F**) The influence on cell proliferation ability of miR-128-3p mimic and miR-128-3p inhibitor was assessed by CCK-8 assay. (**G** and **H**) The protein levels of c-Fos, NFATc1, and Ctsk were analyzed by Western blotting, respectively. (**I**) Transwell assay detecting the migration ability of RAW 264.7 cells (Scale bar: 100 μm). (**J**) Representative images of TRAP-positive osteoclasts formation (Scale bar: 200 μm). ^*^P < 0.05, ^**^P < 0.01; ^***^P < 0.001. Abbreviation: ns: not significant.

We next examined the levels of miR-128-3p in bone tissues. Our results showed that the level of miR-128-3p in the bone tissues from patients with osteoporosis increased significantly (Figure 4A). To further investigate the effects of miR-128-3p on RAW 264.7 cells, miR-128-3p mimics and inhibitors were transfected into RAW 264.7 cells, respectively, and the expression of miR-128-3p after transfection is shown in Figure 4B. CCK-8 assay results indicated that the proliferation of RAW 264.7 cells was promoted by miR-128-3p upregulation, and attenuated by miR-128-3p inhibition (Figure 4F). Transwell assays indicated that miR-128-3p upregulation promoted the migration of RAW 264.7 cells, while the miR-128-3p inhibitor attenuated it (Figure 4I). As for osteoclast differentiation, after RANKL stimulation for 5 days, qRT-PCR and Western blotting showed that the mRNA and protein levels of the osteoclastogenesis markers c-Fos, NFATc1 and Ctsk increased significantly after transfection with miR-128-3p mimic, while they decreased after introducing miR-128-3p inhibitor (Figure 4C–4E, 4G, and 4H). In addition, TRAP staining showed that after we increased the expression of miR-128-3p, the number of TRAP-positive multinucleated osteoclasts significantly increased (Figure 4J). The above results indicated that miR-128-3p promoted the proliferation, migration, and osteoclast differentiation of RAW 264.7 cells.

### miR-128-3p regulates the migration and osteoclast differentiation of RAW 264.7 cells by targeting NFAT5

**Figure 5:**
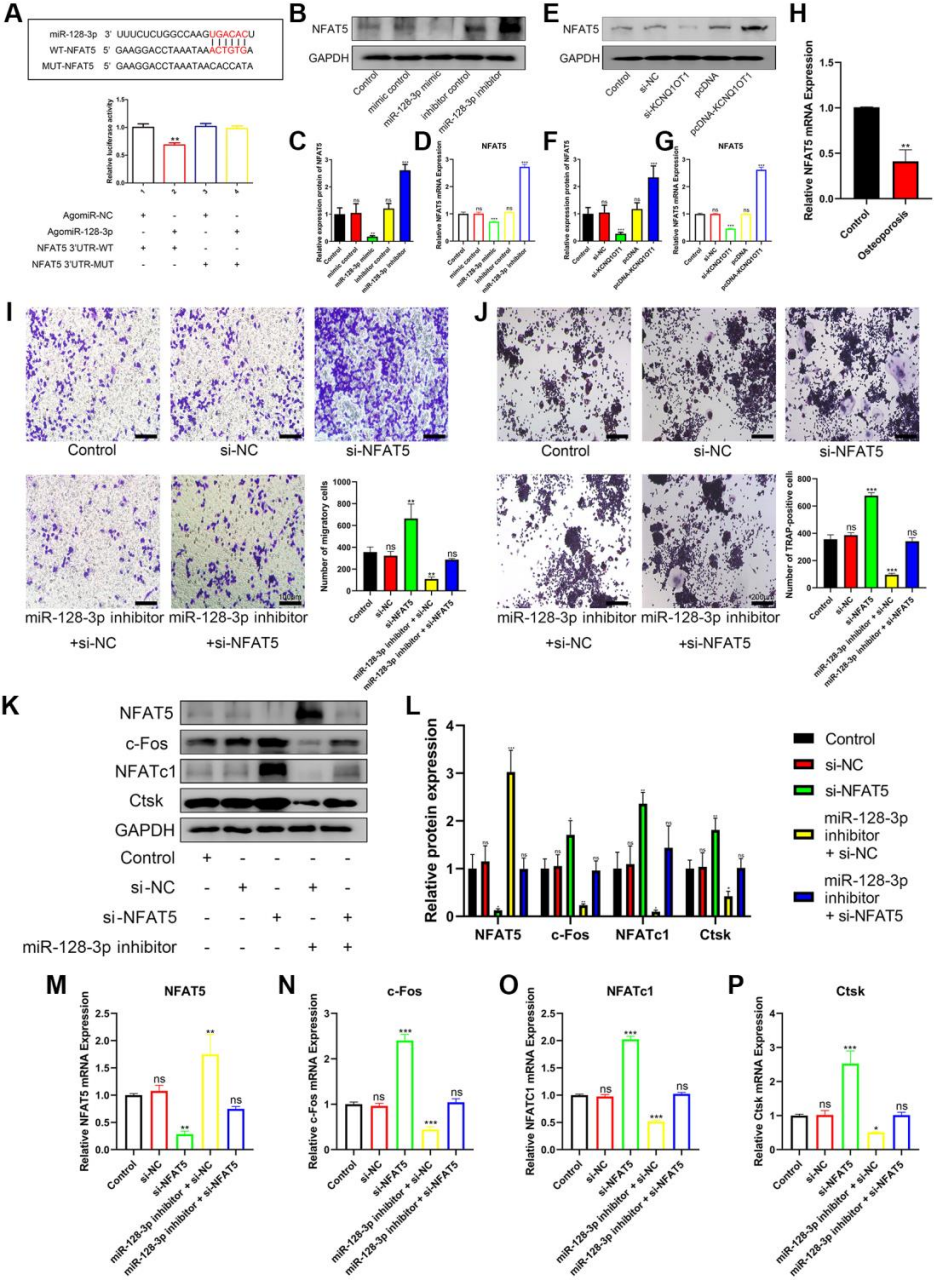
**miR-128-3p regulates the migration and osteoclast differentiation of RAW 264.7 cells by targeting NFAT5.** (**A**) StarBase database showing the putative binding sites between miR-128-3p and NFAT5. The relative luciferase activity was detected in HEK-293T cells transfected with agomiR-NC or agomiR-128-3p in WT and MUT groups, respectively. (**B**–**D**) The mRNA and protein expression of NFAT5 after transfection of miR-128-3p mimic or miR-128-3p inhibitor, were analyzed by qRT-PCR and Western blotting, respectively. (**E**–**G**) The mRNA and protein expression of NFAT5 after transfection of pcDNA-KCNQ1OT1 or si-KCNQ1OT1, were analyzed by qRT-PCR and Western blotting, respectively. (**H**) Relative expression of NFAT5 in non-osteoporotic and osteoporotic bone tissues was detected by qRT-PCR (*N* = 6, 3 osteoporotic and 3 non-osteoporotic). (**I**) The influence on cell migration ability of si-NFAT5 and the rescue effect of miR-128-3p inhibitor was assessed by transwell migration assay (Scale bar: 100 μm). (**J**) Multinucleated osteoclasts were stained by TRAP and then counted (Scale bar: 200 μm). (**K**–**P**) Five days after osteoclastic differentiation, qRT-PCR and Western blotting were performed to detect the mRNA and protein levels of NFAT5, c-Fos, NFATc1, and Ctsk in RAW 264.7 cells transfected with si-NFAT5, miR-128-3p inhibitor or si-NFAT5 + miR-128-3p inhibitor. ^*^P < 0.05, ^**^P < 0.01; ^***^P < 0.001, ns: not significant.

Bioinformatic analysis showed that NFAT5 was a potential target gene for miR-128-3p. Subsequently, dual luciferase assays were performed to investigate the ability of miR-128-3p to directly target NFAT5. As shown in Figure 5A, upregulating miR-128-3p significantly reduced luciferase activity in the NFAT5-UTR-WT group, while there was no significant change in the NFAT5-UTR-MUT group. In addition, qRT-PCR and Western blotting results also showed that the mRNA and protein levels of NFAT5 significantly reduced after transfection of miR-128-3p mimic into RAW 264.7 cells (Figure 5B–5D), were also significantly reduced after transfection with si-KCNQ1OT1 (Figure 5E–5G). Additionally, we found that the mRNA levels of NFAT5 decreased significantly in osteoporotic bone tissues, compared with healthy control tissues (Figure 5H). Then transwell assays were conducted, and compared with other groups, RAW 264.7 cells transfected with si-NFAT5 were enhanced for migration, and the reversed effect was observed when co-transfecting si-NFAT5 with miR-128-3p inhibitor (Figure 5I). It was very obvious that si-NFAT5 decreased the expression of NFAT5, but increased the expression of c-Fos, NFATc1, and Ctsk, which were all rescued after transfection with miR-128-3p inhibitor (Figure 5K–5P). Similarly, TRAP staining showed that si-NFAT5 significantly increased the amount of Trap-positive multinucleated osteoclasts in RAW 264.7 cells, while this effect was weakened when cells were co-transfected with si-NFAT5 and miR-128-3p inhibitor (Figure 5J). Based on the above results, we demonstrated that miR-128-3p could regulated the migration and osteoclast differentiation of RAW 264.7 cells by targeting NFAT5.

### KCNQ1OT1 inhibits osteoclast differentiation by upregulating NFAT5 expression via sponging miR-128-3p

**Figure 6:**
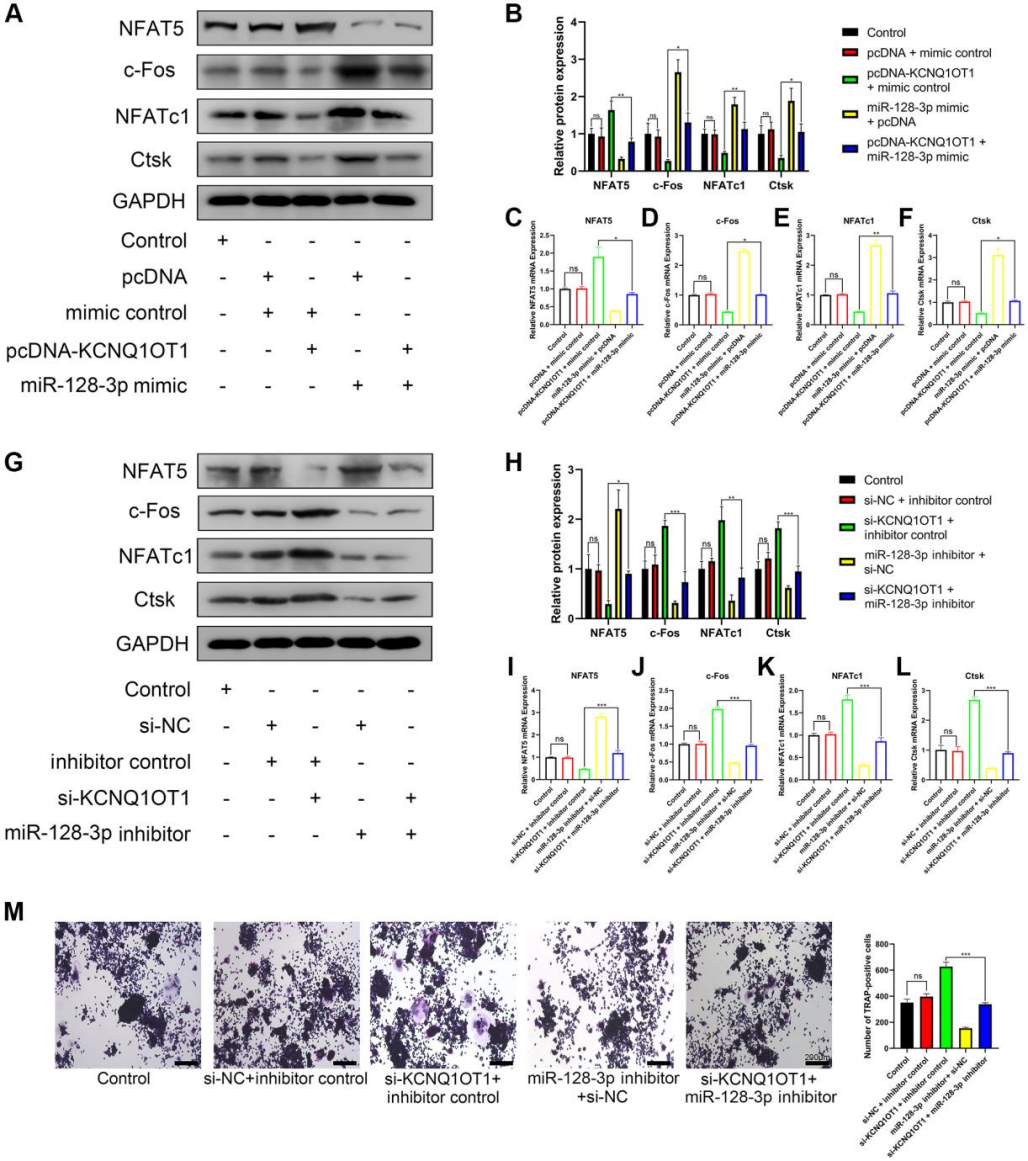
**KCNQ1OT1 inhibits osteoclast differentiation by upregulating NFAT5 expression via sponging miR-128-3p.** (**A**–**F**) RAW 264.7 cells were transfected with pcDNA-KCNQ1OT1, miR-128-3p mimic or pcDNA-KCNQ1OT1 + miR-128-3p mimic respectively, and then treated with RANKL for 5 days. qRT-PCR and Western blotting showed the mRNA and protein expression levels of NFAT5, c-Fos, NFATc1, and Ctsk. (**G**–**L**) Similar experiments were also performed in RAW 264.7 cells transfected with si-KCNQ1OT1, miR-128-3p inhibitor or si-KCNQ1OT1 + miR-128-3p inhibitor. (**M**) Multinucleated osteoclasts were stained by TRAP and then counted (Scale bar: 200 μm). ^*^P < 0.05, ^**^P < 0.01; ^***^P < 0.001, ns: not significant.

Overexpression of KCNQ1OT1 promoted the expression of NFAT5 and inhibited the expression of c-Fos, NFATc1, and Ctsk, which could be rescued using miR-128-3p mimic (Figure 6A–6F). Meanwhile, miR-128-3p inhibitor rescued the effect of si-KCNQ1OT1 (Figure 6G–6L). TRAP staining showed that miR-128-3p inhibitor rescued the effect of si-KCNQ1OT1 on osteoclast differentiation (Figure 6M). Conclusively, KCNQ1OT1 inhibited osteoclast differentiation by regulating the miR-128-3p/NFAT5 axis.

## DISCUSSION

lncRNAs are a key component of ceRNA networks and they mainly regulate cell function through a lncRNA-miRNA-mRNA axis model [15–17]. A number of studies have shown that the lncRNA KCNQ1OT1 can enhance osteoblast viability, promote osteogenic differentiation and inhibit osteolysis. For instance, KCNQ1OT1 promotes MC3T3-E1 cell proliferation, migration, and survival via modulating the miR-701-3p/FGFR3 axis [20]. Wang et al., found that KCNQ1OT1 regulates osteogenic differentiation by activating Smad5 expression via competing for miR-320a [21], while Gao et al., demonstrated that KCNQ1OT1 played a significant role in osteogenic differentiation through Wnt/β-catenin pathway activation [22]. Another study showed that KCNQ1OT1 could ameliorate osteolysis by inhibiting miR-21a-5p [23]. However, the effect of KCNQ1OT1 on osteoclast differentiation, proliferation and migration and its regulating mechanism still needs to be explored. Additionally, it is essential for osteoclast precursors to have the proper abilities for proliferation and migration during the mature process and normal function. Mature osteoclasts play a major role in bone resorption. In light of this, we investigated the association between KCNQ1OT1 and the viability of RAW 264.7 cells. KCNQ1OT1 has been shown to promote bone formation in previous studies, and we speculated that KCNQ1OT1 could inhibit bone resorption. In this study, we observed significantly decreased KCNQ1OT1 expression in osteoporotic bone tissues compared with healthy controls. Upregulation of KCNQ1OT1 significantly inhibited the proliferation and migration of RAW 264.7 cells, while silencing KCNQ1OT1 could enhance their proliferation and migration. In addition, overexpression of KCNQ1OT1 decreased the expression of osteoclastogenesis marker genes and inhibited RANKL-induced osteoclast differentiation.

Using starBase and other databases, we also found that miR-128-3p was a potential target of KCNQ1OT1 and might also regulate the expression of NFAT5. Dual luciferase reporter assays confirmed the interaction relationship between miR-128-3p and KCNQ1OT1, and upregulation of KCNQ1OT1 could significantly downregulate the expression of miR-128-3p. miR-128-3p was found to promote osteoclast formation, inhibit osteoblast differentiation, and fracture healing [29, 33]. We further confirmed that miR-128-3p enhanced the proliferative and migrating ability of RAW 264.7 cells, promoted the osteoclast differentiation induced by RANKL and increased the expression of osteoclastogenesis marker genes. We also found that the expression of miR-128-3p was increased significantly in osteoporotic bone tissues. It has also been shown that NFAT5 can inhibit osteoclastogenesis induced by RANKL [32]. In our further investigation, dual luciferase reporter analysis indicated that NFAT5 was the downstream molecule of miR-128-3p. Meanwhile, NFAT5 expression decreased in patients with osteoporosis. In addition, our experimental results clearly indicated that KCNQ1OT1 could regulate NFAT5 through competitively binding with miR-128-3p and blocking its function. KCNQ1OT1 upregulation significantly promoted NFAT5 expression. Furthermore, miR-128-3p mimic significantly reversed the inhibitory effect of overexpression of KCNQ1OT1 on osteoclast differentiation of RAW 264.7 cells. Moreover, miR-128-3p inhibitor significantly reversed the promoting effect of si-KCNQ1OT1 and si-NFAT5 on osteoclast differentiation. Based on the above experimental results, we found and verified the role of the KCNQ1OT1/miR-128-3p/NFAT5 axis in osteoclast differentiation.

**Figure 7:**
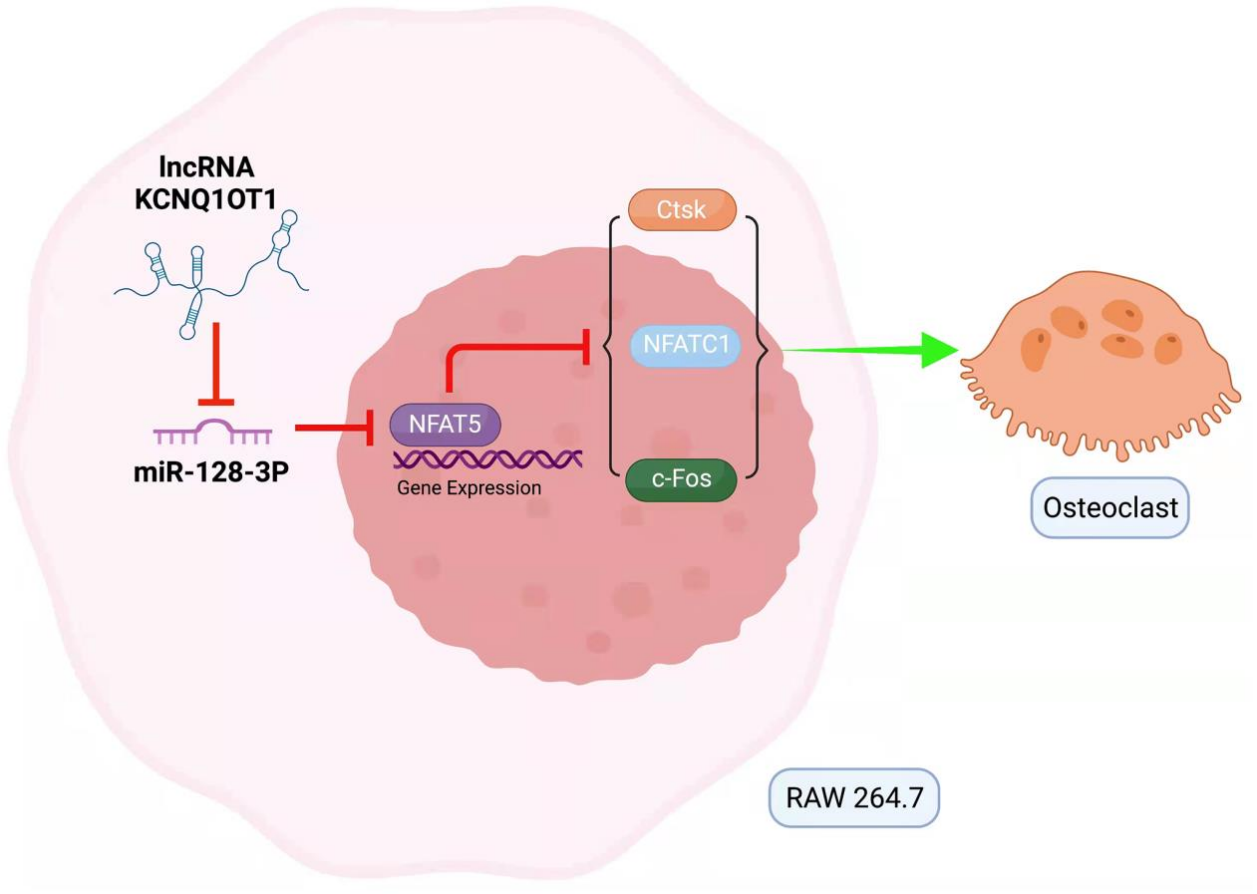
**Mechanism of lncRNA KCNQ1OT1/miR-128-3p/NFAT5 axis mediating osteoclast differentiation.** KCNQ1OT1 competitively binds to miR-128-3p, which leads to upregulation of NFAT5. NFAT5 inhibits the expression of osteoclastogenesis markers such as c-Fos, NFATc1, and Ctsk, thus inhibiting osteoclast differentiation.

In summary, this study suggests that KCNQ1OT1, as a sponge for miR-128-3p, inhibits the osteoclast differentiation of RAW 264.7 cells through NFAT5 activation (Figure 7), which might provide a promising therapeutic approach to the prevention and treatment of osteoporosis.

## MATERIALS AND METHODS

### Bioinformatic analysis

StarBase 2.0 (http://starbase.sysu.edu.cn/) was used to predict target miRNAs for KCNQ1OT1. The upstream miRNAs that might regulate NFAT5 were predicted by three bioinformatics databases: starBase, miRwalk (http://mirwalk.umm.uni-heidelberg.de/) and TargetScan (http://www.targetscan.org/). A Venn diagram was used to display the intersection of the results from the databases mentioned above. The intersections in the Venn diagrams represented the miRNAs of interest.

### Sample collection

The bone tissue samples analyzed in this study were obtained from 3 patients with osteoporosis (61.0 ± 4.4 years) and 3 patients without osteoporosis (58.7 ± 4.5 years) who underwent lumbar surgery in the Qilu Hospital of Shandong University from September 2021 to November 2021. The patients with osteoporosis were defined as having a bone mineral density (BMD) measured by dual-energy X-ray absorptiometry with a T score was less than −2.5. This study was approved by the Medical Ethics Committee of Qilu Hospital of Shandong University, and all participants signed informed consent prior to enrollment in this study.

### Cell culture

RAW 264.7 and HEK-293T cells, purchased from the Cell Bank of Chinese Academy of Sciences, were cultured in DMEM supplemented with 10% fetal bovine serum and 1% penicillin/streptomycin. All cell lines were cultured at 37°C with 5% CO_2_.

In order to induce osteoclast differentiation, RAW 264.7 cells were incubated with RANKL (100 ng/mL; Novoprotein Scientific, Shanghai, China) for 5 days.

### qRT-PCR

Total RNA was extracted using TRIzol (Ambion, CA, USA) were reverse-transcribed into cDNAs using the M-MLV RTase cDNA Synthesis Kit (Takara, Japan) according to the manufacturer's instructions. The expression level of RNA was detected by qRT-PCR using AceQ qPCR SYBR Green Master Mix (Takara, Japan), analyzed on a Mx4000 Multiplex Quantitative PCR System (Stratagene, La Jolla, CA, US) and calculated using the 2^−ΔΔCT^ method. The primers in this study were synthesized by GenePharma (Shanghai, China), and the primer sequences used in this study were: KCNQ1OT1 forward, 5′-TGG GGA AGG CTG CTT ATT CG-3′, reverse, 5′-CGG AAC CAC TGT AGA CCC AC-3′; miR-128-3p forward, 5′-CGA GCG AAG TGC TGT CAT AG-3′, reverse, 5′-CAG TGC GTG TCG TGG AGT-3′; NFAT5 forward, 5′-ACT TGC TGG ATA ACA GTC GGA-3′, reverse, 5′-ATC TCG TCG TTT GAC CCC C-3′; NFATc1 forward, 5′-CTG CAA CAA GCG CAA GTA CA-3′, reverse, 5′-AGG TCC AGA GTG CTA TCG GT-3′; c-Fos forward, 5′-AGA GCG GGA ATG GTG AAG AC-3′, reverse, 5′-AGT TGA TCT GTC TCC GCT TGG-3′; Ctsk forward, 5′-AGC AGA ACG GAG GCA TTG AC-3′, reverse, 5′-ATT TAG CTG CCT TTG CCG TG-3′; GAPDH forward, 5′-CAC UCA AGA UUG UCA GCA ATT-3′, reverse, 5′-UUG CUG ACA AUC UUG AGU GAG-3′; and U6 forward, 5′-CAG CAC ATA TAC TAA AAT TGG AAC G-3′, reverse, 5′-ACG AAT TTG CGT GTC ATC C-3′.

### Western blotting

We prepared cell lysates on ice using RIPA dissolution buffer. Then protein samples were added with loading buffer (5×), separated by electrophoresis in sodium dodecyl sulphate–polyacrylamide gels, transferred to polyvinylidenedifluoride (PVDF) membranes, and incubated with the following primary antibodies: rabbit polyclonal anti-NFAT5 (1:1000, Abcam, #ab3446, Cambridge, UK), rabbit monoclonal anti-NFATc1 (1:1000, CST, #8032), mouse monoclonal anti-c-Fos (1:1000, Abcam, #ab208942), rabbit polyclonal anti-Ctsk (1:1000, Abcam, #ab19027) and rabbit monoclonal anti-GAPDH (1:1000, CST, #2118). After incubation with the appropriate secondary antibody, the protein bands were observed using an electrochemiluminescence reagent and analyzed with ImageJ.

### CCK-8 assays

For this, cells were seeded into 96-well plates (2 × 10^3^ cells/well). Cell proliferation was examined every 24 hours: 10 μL of CCK-8 solution was added to each well, followed by incubation at 37°C for 2 h, and the optical density at 450 nm was finally measured using microplate reader.

### Transwell migration assays

RAW 264.7 cells (4.0 × 10^4^ cells/well) were seeded into the upper chambers of transwells in serum-free medium, and 700 μL of complete medium was added to the lower chamber as a chemical attractant. After incubation at 37°C for 24 h, cells on the upper surface were removed, and cells which migrated to the lower surface of these membranes were stained with 0.1% crystal violet solution and analyzed.

### TRAP staining

A TRAP staining kit (BestBio, China) was used according to the manufacturer's instructions. Cells were first fixed with the fixative solution in the kit and washed with distilled water. Subsequently, the substrate incubation solution was added and samples were incubated at 37°C for 60 minutes in the dark. Finally, cells were counterstained with hematoxylin. TRAP-positive multinucleated cells containing three or more nuclei were counted, which were considered osteoclasts.

### Cell transfection

KCNQ1OT1 overexpression vector (pcDNA-KCNQ1OT1) and empty plasmid, the small interfering RNA (siRNA) against KCNQ1OT1 (si-KCNQ1OT1) and NFAT5 (si-NFAT5) and their negative controls (si-NC), the miR-128-3p mimic, miR-128-3p inhibitor and corresponding negative controls were all synthesized at GenePharma (Shanghai, China). The reagents mentioned above were transfected or co-transfected into RAW 264.7 cells using Lipofectamine 2000 (Thermo Fisher Scientific, Waltham, MA, USA). The sequences were as follows: si-KCNQ1OT1 (5′-GCC GAA GAG ACA UCU UAA ATT-3′); si-NFAT5 (5′-GCC CAU GCA AUU UCA GAA UTT-3′).

### Dual luciferase reporter assays

HEK-293T cells were co-transfected with dual luciferase reporter gene constructs (the wild type or mutant sequences of KCNQ1OT1 or NFAT5) and miR-128-3p mimic or negative control (NC), respectively. After 24 h, luciferase activity was determined using a Dual Luciferase Reporter Assay System (Promega, Madison, WI, USA).

### Statistical analysis

All continuous data were expressed as the mean ± standard deviation (SD) and analyzed using GraphPad Prism 8.0 (GraphPad Inc, La Jolla, CA, USA). Student’s *t*-test was used to evaluate the differences between two groups, and the differences between more than two groups were analyzed by one-way analysis of variance (ANOVA). All experiments were repeated at least three times. *P* < 0.05 was considered statistically significant.
